# Is there concordance between bone and tendon cultures in patients with foot tissue loss?

**DOI:** 10.1590/1677-5449.190063

**Published:** 2019-11-08

**Authors:** Vanessa Prado dos Santos, Carlos Alberto Silveira Alves, André Brito Queiroz, Maria Goreth Matos de Andrade Barberino, Ronald José Ribeiro Fidelis, Cícero Fidelis, José Siqueira de Araújo

**Affiliations:** 1 Universidade Federal da Bahia – UFBA, Instituto de Humanidades Artes e Ciências Professor Milton Santos, Salvador, BA, Brasil.; 2 Universidade Federal da Bahia – UFBA, Complexo Hospitalar Universitário Professor Edgard Santos, Salvador, BA, Brasil.; 3 Universidade Federal da Bahia – UFBA, Faculdade de Medicina da Bahia, Salvador, BA, Brasil.

**Keywords:** microbiological analysis, wound infections, diabetic foot

## Abstract

**Background:**

Deep infections of the extremities are a challenge that threaten limb salvage.

**Objectives:**

To investigate whether the results of bone and deep tissue cultures from patients with trophic limb ulcers coincide.

**Methods:**

A retrospective study was conducted with data from 54 patients with deep trophic limb ulcers admitted to the Complexo Hospitalar Universitário Professor Edgard Santos, Salvador (BA), Brazil. The study analyzed all patients for whom cultures of material from foot wounds in patients with tissue loss had been performed using two specimen types: bone and fragments of deep tendon. The study analyzed concordance between the two sample types and total number of microorganisms and numbers of microorganisms by Gram staining in both samples.

**Results:**

The mean age of the 54 patients in the sample was 63.6 years, 80% had PAOD, 70% were diabetic, and 72% were hypertensive. Analysis of the cultures showed that 28 (52%) pairs of samples from the 54 patients exhibited complete concordance, with the same microorganisms grown from fragments of deep tendon and bone. There was partial disagreement in 13 samples (24%) and total disagreement in 13 (24%). On average, 1.62 microorganisms were isolated from deep tendon fragments and 1.72 were isolated from bone samples. Analyzing Gram-positive microorganisms separately, the mean number of species grown was 0.48 for tendon cultures and 0.44 for bone cultures. In contrast, the mean number of Gram-negative microorganisms isolated was 1.14 for tendon samples and 1.27 for bone samples.

**Conclusions:**

Around half of the patients with foot tissue loss had bone and tendon cultures that coincided exactly.

## INTRODUCTION

In Brazil, the prevalence of peripheral arterial occlusive disease (PAOD) is around 10% among diabetics and 2.6% among non-diabetic patients.^1^ Diabetes mellitus (DM) is considered one of the main risk factors for peripheral vascular disease.^2^ Patients with critical limb ischemia, characterized by pain at rest and ulcer or gangrene secondary to peripheral vascular disease are at high risk of cardiovascular events and limb loss.^2^ The risk of limb loss increases in the presence of infection and major amputations performed on diabetic patients are frequently associated with PAOD and/or infection.^3^^,^ 4 Diabetic patients with infected ischemic wounds are up to 90 times more likely to undergo a lower limb amputation than those without ischemia or infection.^5^

Deep tissue cultures are recommended to guide treatment of the infected ulcers of diabetic and/or ischemic patients with severe limb wounds, in order to determine microbiological diagnosis and plot antibiograms, helping to indicate the most appropriate specific treatment.^6^ In patients with indications for surgical treatment, the culture should be collected after removal of nonviable tissues and if there is bone involvement it may be necessary to increase the duration of antibiotic therapy.^6^^,^ 7

Research in the literature shows that a range of different culture methods are used for identification of the infectious agent, including curettage, aspiration, biopsy, and even swabs, although the last of these is discouraged by many authors.^6^^,^ 8 Comparisons of the microbial flora isolated from surface and deep cultures of infected wounds show that the results of deep cultures differ from those of surface samples and studies emphasize the superiority of deep tissue cultures for identification of the pathogen responsible for infection.^9^^,^ 10 Ideally, the material collected should contain deep tissue, to avoid culturing strains that have colonized the ulcer but are not the cause of the infectious condition.^11^ However, there are few studies reporting comparisons of the results of cultures of material from different types of deep tissue. The objective of this study was therefore to investigate whether there was concordance between the results of cultures of bone and deep tissues from patients with extensive trophic ulcers requiring surgical treatment.

## METHODS

A retrospective, descriptive study was conducted with data from 54 patients with tissue loss or gangrene admitted to the Complexo Hospitalar Universitário Professor Edgard Santos, run by the Universidade Federal da Bahia (UFBA), Salvador, BA, Brazil. The study included all consecutive patients for whom cultures of foot tissue loss had been performed using both methods of collecting specimens (bone fragments and deep tendon fragments) for cultures and antibiograms, which were conducted by the microbiology laboratory at the same institution. In all patients, material was collected for cultures during surgical treatment (deep debridement or minor amputation). Material was collected after removal of all tissues that were macroscopically compromised and sent for cultures separately.

Patient characteristics (sex, age, and presence of systemic arterial hypertension, DM, and PAOD) and concordance between the cultures conducted with the two different types of sample (bone and deep tendon) were analyzed. Additionally, a comparative analysis was conducted of the total number of microorganisms and the numbers of Gram positive and Gram negative microorganisms isolated from bone and deep tendon samples.

Patient data were collected from patient medical records, clinical follow-up charts, and culture records archived at the vascular surgery service and microbiology laboratory at the Complexo Hospitalar Universitário Professor Edgard Santos.

The research project was approved by the Research Ethics Committee at the Complexo Hospitalar Universitário Professor Edgard Santos (protocol number 33051514.0.0000.0049).

Statistical analysis of data was conducted using Epi-Info, version 3.3.2, from February 2005. Categorical (qualitative) variables were studied using frequency tables and continuous (quantitative) variables were expressed as summary measures, such as mean and standard deviation. The chi-square test was used for comparative analyses of qualitative variables. Means of variables expressed numerically (quantitative) were compared using analysis of variance (ANOVA). We adopted a significance level of 5% (p ≤ 0.05) for defining statistical differences between groups, in terms of the study variables.

## RESULTS

A total of 54 patients were included consecutively in the sample. Mean age was 63.6 (±14.66) years and 50% were male. The majority of patients (80%) had PAOD associated with tissue loss of the extremities. With regard to comorbidities, 70% were diabetics and 72% were hypertensive. According to the Rutherford classification, 83% of the lesions were Category 5, and 17% were Category 6. Patients’ characteristics are summarized in Table 1. Analysis of the full results for bone and deep tendon cultures from the 54 patients in the sample revealed that in 28 cases (52%) the samples coincided completely, i.e. were identical, with the same microorganisms cultured from the deep tendon fragments and the bone fragments. There was partial mismatch in 13 cases (24%) and total disagreement in the remaining 13 (24%). Five (9%) deep tendon cultures were negative and all of the bone cultures were positive. With regard to the number of microorganisms cultivated, in 43% of the bone cultures and 41% of the deep tendon cultures, just one species of microorganism grew (Table 2). Comparison between the results of the cultures revealed that an average of 1.62 microorganisms grew from the deep tendon fragments and 1.72 from the bone samples. Analyzing microorganisms separately by Gram staining result, the mean numbers of Gram-positive species cultivated from tendons and bone tissues were 0.48 and 0.44, respectively. In contrast, for the Gram-negative microorganisms, the mean number of microorganisms cultivated was 1.14 for tendon samples and 1.27 for bone samples (Table 3).

**Table 1:** Characteristics of 54 patients with deep trophic ulcers included in the study and concordance between cultures from samples of tendon and bone.

**Characteristics of the patient sample (n = 54)**	**n (%)**
Male	27 (50%)
Mean age	63.6 (±14.6) years
Mean white blood cell count	10,932 leukocytes/mm^3^
History of heart disease	10 (19%)
Diabetes mellitus	38 (70%)
Systemic arterial hypertension	79 (72%)
Current smoking	24 (44%)
Chronic renal failure	4 (7%)
PAOD	43 (80%)
Rutherford Classification	45 (83%)
Category 5	
Category 6	9 (17%)
Agreement between cultures (bone and deep tendon)	
Complete agreement / identical	28 (52%)
Partial agreement	13 (24%)
No agreement/ total disagreement	13 (24%)

**Table 2:** Comparative analysis of numbers of microorganisms grown in the 54 cultures.

**Culture results (n = 54)**	**Sample cultured**		
	**Bone**	**Tendon**	
	**n (%)**	**n (%)**	
Negative cultures	0 (0%)	5 (9%)	
Cultures with a single species of microorganism	23 (43%)	22 (41%)	
Cultures with two species of microorganism	23 (43%)	17 (31%)	p = 0.09
Cultures with three species of microorganism	8 (14%)	8 (15%)	
Cultures with four species of microorganism	0 (0%)	2 (4%)	

**Table 3:** Comparative analysis of samples with results of cultures from the 54 patients studied.

**Culture results (n = 54)**	**Sample cultured**		**p**
	**Bone**	**Tendon**	
Number of microorganisms grown, mean (±SD)	1.72 (±0.71)	1.62 (±0.97)	0.57
Number of Gram-positive microorganisms, mean (±SD)	0.44 (±0.63)	0.48 (±0.66)	0.76
Number of Gram-negative microorganisms, mean (±SD)	1.27 (±0.78)	1.14 (±0.91)	0.43
Presence of Gram-positive species, n (%)	20 (37%)	21(39%)	0.42
Presence of Gram-negative species, n (%)	45 (83%)	40 (74%)	0.12

SD = standard deviation.

## DISCUSSION

Our study compared the results of cultures of deep specimens collected in a surgery unit after removal of nonviable tissues and found some degree of agreement, total or partial, in 76% of cases studied. Other authors have compared cultures of different sample types, with the most common comparison being between superficial and deep tissues. We believe that it is important to compare cultures of bone and deep tendon, because treatment may be changed if bone infection is present.^7^ Kessler et al.^10^ compared cultures from material collected using swabs or needle puncture from 21 diabetic patients with lower limb ulcers, finding identical results in four patients. Senneville et al.^12^ studied concordance between cultures by ulcer swab and percutaneous bone biopsy, demonstrating that cultures were identical in 17.4%, of 69 patients. In a different study, with 31 patients, Senneville et al.^13^ compared material collected by needle puncture and transcutaneous bone biopsy, finding that 32.3% of results were identical. In our study, with 54 patients, we observed that 52% of cultures had identical results. The higher percentage of total agreement is probably because we compared two specimens collected from deep tissues (bone and tendon) during a surgical procedure.

Kessler et al.^10^ found a mean of 1.09 different microorganisms isolated from material collected by deep puncture from diabetic patients who did not require surgical treatment. In the two articles by Senneville et al.,^12^^,^ 13 the mean number of microorganisms isolated in bone biopsy samples was 1.54 and 1.35, whereas we found a mean of 1.72 microorganisms in bone cultures and 1.62 in tendon cultures; both of these means are higher than reported by other authors. However, our patients also differ from others in terms of the depth of lesions, the need for surgical treatment in all cases, the extensive tissue loss, and the high prevalence of associated PAOD. We had around 40% of single microbe cultures, which is similar to Kessler et al.,^10^ who collected material by needle puncture from non-surgical patients with diabetic ulcers, finding 48% of single microbe cultures. The predominance of Gram-negative microorganisms in our cultures of deep tissue samples, both of bone and of tendon, differs from results observed by other authors, possibly because of the severity and depth of our patients’ lesions.^10^^,^ 12 ^,^ 13 However, a literature review showed an increase in the prevalence of Gram-negative microorganisms in deep diabetic foot ulcers in studies undertaken in different countries.^14^ In Brazil, a study conducted with 141 patients with diabetic ulcers, with cultures of material collected by swab, found that Gram-negative bacilli were the most frequent microorganisms.^15^ Also in Brazil, among 78 patients who underwent major amputations because of infected diabetic feet, Cardoso et al.^16^ found that Gram-negative microorganisms were among the most frequently grown in cultures of deep tissues.

Our study is subject to the limitations inherent to a retrospective study, but it contributes to the literature by conducting a comparative analysis of cultures from deep tissues, collected in a surgical unit from patients with extensive and severe lesions, studying the extent to which the results coincide, and also the number and characteristics of the microorganisms found.

## CONCLUSIONS

Considering the results of cultures of specimens collected from different types of deep tissues, in around half of the cases there was total agreement, with identical culture results for bone and tendon from foot tissue loss. The fact that there were discrepancies between the results of many pairs of cultures from two different deep tissues (tendons and bones) suggests that, whenever possible, specimens of both materials should be collected.
