# Supplementary material for: Is there concordance between bone and tendon cultures in patients with foot tissue loss?
Source: J Vasc Bras. 2019 Nov 8;18:e20190063. doi: 10.1590/1677-5449.190063 (PMC6852700; doi:10.1590/1677-5449.190063)
Supplement: Translation

# Translation: Existe concordância entre as culturas de osso e tendão em pacientes com lesões profundas de extremidades?

**Authors:** Vanessa Prado dos Santos, Carlos Alberto Silveira Alves, André Brito Queiroz, Maria Goreth Matos de Andrade Barberino, Ronald José Ribeiro Fidelis, Cícero Fidelis, José Siqueira de Araújo

**Affiliations:** 1 Universidade Federal da Bahia – UFBA, Instituto de Humanidades Artes e Ciências Professor Milton Santos, Salvador, BA, Brasil.; 2 Universidade Federal da Bahia – UFBA, Complexo Hospitalar Universitário Professor Edgard Santos, Salvador, BA, Brasil.; 3 Universidade Federal da Bahia – UFBA, Faculdade de Medicina da Bahia, Salvador, BA, Brasil.

**Keywords:** análise microbiológica, infecção de feridas, pé diabético

## Abstract

**Contexto:**

As infecções profundas de extremidades representam um desafio para o salvamento de membro dos pacientes.

**Objetivos:**

Investigar se existe concordância entre as culturas de osso e tecido profundo em pacientes com lesões tróficas de extremidades.

**Métodos:**

Foi realizado um estudo retrospectivo incluindo 54 pacientes com lesões tróficas profundas de extremidades internados, no Complexo Hospitalar Universitário Professor Edgard Santos, Salvador (BA), Brasil. Foram incluídos todos os pacientes que realizaram culturas de lesões tróficas profundas, com duas modalidades de coleta de exame: osso e fragmentos de tendão profundo. Analisaram-se a concordância, o número total de microrganismos e o número de microrganismos de acordo com a coloração de Gram em ambas as amostras.

**Resultados:**

Entre os 54 doentes incluídos na amostra, a média de idade foi de 63,6 anos, 80% apresentavam DAOP, 70% eram diabéticos, e 72% hipertensos. Estudando as culturas dos 54 pacientes, 28 amostras (52%) foram completamente concordantes, sendo cultivados os mesmos microrganismos nos fragmentos de tendão profundo e de osso. Houve discordância parcial em 13 amostras (24%), e discordância total em 13 (24%). Observou-se que cresceu em média 1,62 microrganismo nos fragmentos de tendão profundo, e 1,72 nas amostras de osso. Analisando separadamente os microrganismos gram-positivos, a média de espécies cultivadas foi de 0,48 em tendão e de 0,44 em tecido ósseo. Por outro lado, para os microrganismos gram-negativos, a média de microrganismos cultivados foi de 1,14 e 1,27 nas amostras de tendão e de osso, respectivamente.

**Conclusões:**

Cerca de metade dos pacientes portadores de lesões tróficas profundas de extremidades apresentaram concordância total entre as culturas de osso e de tendão.

## INTRODUÇÃO

No Brasil, a prevalência de doença arterial obstrutiva periférica (DAOP) é de cerca de 10% entre diabéticos e de 2,6% entre pacientes não diabéticos^1^. O diabetes melito (DM) é considerado um dos principais fatores de risco para a doença vascular periférica^2^. Pacientes com isquemia crítica de membro, caracterizada por dor ao repouso, úlcera ou gangrena, secundária à doença vascular periférica têm um alto risco de eventos cardiovasculares e de perda do membro^2^. O risco de perda do membro aumenta na presença de infecção, sendo que as amputações maiores em doentes diabéticos frequentemente estão associadas à presença de DAOP e/ou infecção^3^^,^ 4. Pacientes diabéticos com lesões isquêmicas e infectadas têm até 90 vezes mais chance de sofrer uma amputação de membro inferior do que aqueles sem isquemia ou infecção^5^.

Para orientar o tratamento das lesões infectadas de doentes diabéticos e/ou isquêmicos com lesões graves de extremidades, a realização de cultura de tecido profundo está indicada, visando estabelecer o diagnóstico microbiológico e a realização do antibiograma, guiando a terapêutica específica apropriada^6^. Nos pacientes com indicação de tratamento cirúrgico, a cultura deve ser coletada após a retirada dos tecidos desvitalizados e, havendo comprometimento ósseo, pode ser necessário ampliar o tempo de administração da antibioticoterapia^6^^,^ 7.

Na literatura, pesquisas mostram que distintos métodos de cultura são utilizados para a identificação do agente infeccioso, como curetagem, aspiração, biópsia e mesmo *swab*s, apesar de estes últimos serem desencorajados por muitos autores^6^^,^ 8. A comparação entre a flora microbiana isolada em culturas superficiais e profundas de lesões infectadas mostrou que o resultado da cultura profunda diferiu daquele da amostra superficial, destacando a superioridade da coleta de cultura de tecido profundo na identificação do patógeno responsável pela infecção^9^^,^ 10. Idealmente, o material coletado deve conter tecido profundo, evitando o crescimento de cepas colonizadoras da úlcera e não causadoras do quadro infeccioso^11^. Porém, poucos estudos trazem comparações entre culturas realizadas a partir de material coletado de tecidos profundos distintos. O objetivo deste estudo foi investigar se existe concordância entre as culturas de osso e tecido profundo em pacientes com lesões tróficas extensas, que necessitam de tratamento cirúrgico.

## MÉTODOS

Foi realizado um estudo descritivo e retrospectivo incluindo 54 pacientes com lesões tróficas profundas de extremidades internados no Complexo Hospitalar Universitário Professor Edgard Santos da Universidade Federal da Bahia (UFBA), Salvador, BA, Brasil. Foram incluídos, consecutivamente, todos os pacientes que realizaram culturas de lesões tróficas profundas com as duas modalidades de material para exame (fragmento de osso e fragmento de tendão profundo) para a realização de cultura e antibiograma no Laboratório do Serviço de Microbiologia da mesma instituição. Todos os pacientes realizaram a coleta de material para a cultura durante o tratamento cirúrgico (desbridamento profundo ou amputação menor). O material foi coletado após a retirada de todo o tecido macroscopicamente comprometido e enviado separadamente para a cultura.

Foram avaliadas as características dos pacientes (sexo, idade e presença de hipertensão arterial sistêmica, DM e DAOP) e a concordância entre as culturas realizadas a partir dos dois tipos de amostra (osso e tendão profundo). Além disso, foram analisadas comparativamente as amostras de osso e tendão profundo quanto ao número de microrganismos cultivados e a presença e o número de microrganismos de acordo com a coloração de Gram.

A coleta de dados dos pacientes foi realizada em prontuários, fichas de acompanhamento clínico e culturas arquivadas no Serviço de Cirurgia Vascular e no Laboratório do Serviço de Microbiologia do Complexo Hospitalar Universitário Professor Edgard Santos.

O projeto de pesquisa foi aprovado no Comitê de Ética em Pesquisa do Complexo Hospitalar Universitário Professor Edgard Santos (protocolo número 33051514.0.0000.0049).

Para análise estatística dos dados, foi utilizado o programa Epi-Info, versão 3.3.2, de fevereiro de 2005. As variáveis categorizadas (qualitativas) foram estudadas por tabelas de frequência, e as variáveis contínuas (quantitativas) por medidas resumo, como média e desvio padrão. Para a análise comparativa das variáveis qualitativas, utilizamos o teste do qui-quadrado. As variáveis expressas numericamente (quantitativas) tiveram suas médias comparadas pela análise de variância (ANOVA). Adotamos o nível de significância de 5% (p ≤ 0,05) para considerar a diferença estatística entre os grupos, em relação às variáveis estudadas.

## RESULTADOS

Foram incluídos consecutivamente na amostra 54 doentes com média de idade de 63,6 (±14,66) anos, sendo que 50% eram do sexo masculino. A maioria dos pacientes (80%) apresentava DAOP associada ao quadro de lesão trófica profunda de extremidade. Quanto às comorbidades, 70% dos doentes eram diabéticos, e 72% hipertensos. Quanto à classificação de Rutherford, 83% das lesões se encontravam na Categoria 5, e 17% na Categoria 6. As características dos pacientes se encontram resumidas na Tabela 1. Quanto aos os resultados do total das culturas de osso e tendão profundo dos 54 pacientes, observou-se que, em 28 casos (52%), as amostras foram completamente concordantes, idênticas, sendo cultivados os mesmos microrganismos nos fragmentos de tendão profundo e de osso. Houve discordância parcial em 13 casos (24%), e discordância total em outros 13 (24%). Cinco culturas (9%) de tendão profundo foram negativas, e todas as culturas ósseas foram positivas. Quanto ao número de microrganismos cultivados, em 43% das culturas de osso e em 41% das culturas de tendão profundo houve o crescimento de apenas uma espécie de microrganismo (Tabela 2). Na comparação entre os resultados das culturas, observou-se que cresceu em média 1,62 microrganismo nos fragmentos de tendão profundo, e 1,72 nas amostras de osso. Analisando separadamente os microrganismos pela coloração de Gram, entre os gram-positivos a média de espécies cultivadas em tendão e em tecido ósseo foi de 0,48 e 0,44, respectivamente. Por outro lado, para os microrganismos gram-negativos, a média de microrganismos cultivados foi de 1,14 nas amostras de tendão e de 1,27 nas amostras de osso (Tabela 3).

**Tabela 1:** Características dos 54 doentes com lesões tróficas profundas incluídos no estudo e concordância entre culturas das amostras de tendão e osso.

**Características da amostra (n = 54)**	**n (%)**
Sexo masculino	27 (50%)
Média de idade	63,6 (±14,6) anos
Média do leucograma	10.932 leucócitos/mm^3^
História de cardiopatia	10 (19%)
Diabetes melito	38 (70%)
Hipertensão arterial sistêmica	79 (72%)
Tabagismo atual	24 (44%)
Insuficiência renal crônica	4 (7%)
Portadores de DAOP	43 (80%)
Classificação de Rutherford	
Categoria 5	45 (83%)
Categoria 6	9 (17%)
Concordância entre culturas (osso e tendão profundo)	
Concordância total/ idênticas	28 (52%)
Concordância parcial	13 (24%)
Sem concordância/ discordância total	13 (24%)

**Tabela 2:** Análise comparativa do número de microrganismos cultivados nas 54 culturas.

**Resultados das culturas (n = 54)**	**Amostra cultivada**		
	**Osso**	**Tendão**	
	**n (%)**	**n (%)**	
Culturas negativas	0 (0%)	5 (9%)	
Culturas com uma espécie de microrganismo	23 (43%)	22 (41%)	
Culturas com duas espécies de microrganismos	23 (43%)	17 (31%)	p = 0,09
Culturas com três espécies de microrganismos	8 (14%)	8 (15%)	
Culturas com quatro espécies de microrganismos	0 (0%)	2 (4%)	

**Tabela 3:** Análise comparativa das amostras com o resultado das culturas dos 54 pacientes estudados.

**Resultados das culturas (n = 54)**	**Amostra cultivada**		**p**
	**Osso**	**Tendão**	
Número de microrganismos cultivados, média (±DP)	1,72 (±0,71)	1,62 (±0,97)	0,57
Número de microrganismos gram-positivos, média (±DP)	0,44 (±0,63)	0,48 (±0,66)	0,76
Número de microrganismos gram-negativos, média (±DP)	1,27 (±0,78)	1,14 (±0,91)	0,43
Presença de espécies gram-positivas, n (%)	20 (37%)	21(39%)	0,42
Presença de espécies gram-negativas, n (%)	45 (83%)	40 (74%)	0,12

DP = desvio padrão.

## DISCUSSÃO

Nossa pesquisa comparou os resultados de culturas de material profundo coletadas em centro cirúrgico após a retirada do tecido desvitalizado, e encontrou alguma concordância, total ou parcial, em 76% dos casos estudados. Outros autores compararam a cultura de diferentes materiais, sendo mais frequente a comparação entre as culturas de material superficial e profundo. Consideramos importante comparar as culturas de osso e tendão profundo, pois o tratamento pode ser alterado na presença de infecção óssea^7^. Kessler et al.^10^ compararam as culturas de material coletado por meio de *swab* e de punção por agulha em 21 pacientes diabéticos com úlcera de membro inferior, encontrando resultados idênticos em quatro pacientes. Senneville et al. estudaram a concordância entre culturas coletadas por meio de *swab* da úlcera e biópsia percutânea do osso, demonstrando que, em 17,4%, dos 69 pacientes, as culturas foram idênticas^12^. Em outro trabalho com 31 pacientes, Senneville et al.^13^ compararam material coletado por meio de punção por agulha e biópsia óssea transcutânea, encontrando 32,3% de resultados idênticos. Na nossa pesquisa, com 54 pacientes, observamos que 52% das culturas tiveram resultados idênticos. Nossa maior porcentagem de concordância total provavelmente se deve à comparação de dois materiais coletados a partir de tecidos profundos (osso e tendão) durante o procedimento cirúrgico.

Kessler et al.^10^ encontraram uma média de 1,09 microrganismo isolado no material coletado por meio de punção profunda em pacientes diabéticos que não necessitavam de tratamento cirúrgico. Nos dois artigos de Senneville et al.^12^^,^ 13, as médias dos microrganismos isolados em amostras de biópsia óssea foram de 1,54 e 1,35, enquanto nós obtivemos uma média de 1,72 em material ósseo e 1,62 em tendão, médias superiores às dos demais autores. No entanto, nossa casuística difere das demais no que concerne à profundidade da lesão, necessidade de tratamento cirúrgico em todos os casos, perda tecidual extensa e alta prevalência de DAOP associada. Obtivemos cerca de 40% de culturas monomicrobianas, resultado semelhante ao de Kessler et al.^10^ que, em pacientes não cirúrgicos com úlceras diabéticas, encontraram 48% de culturas monomicrobianas em material coletado por meio de punção por agulha. A predominância de microrganismos gram-negativos nas nossas culturas de tecido profundo, tanto de osso como de tendão, difere dos resultados encontrados por outros autores, possivelmente devido à gravidade e à profundidade das lesões dos nossos pacientes^10^^,^ 12 ^,^ 13. No entanto, uma revisão da literatura mostrou o aumento da prevalência de microrganismos gram-negativos em lesões profundas do pé diabético em estudos realizados em diferentes países^14^. No Brasil, estudo realizado com 141 pacientes portadores de úlceras diabéticas, com culturas realizadas em material coletado por *swab*, encontrou como microrganismos mais frequentes os bacilos gram-negativos^15^. Também no Brasil, em 78 pacientes submetidos a amputações maiores por pé diabético infectado, Cardoso et al.^16^ encontraram os microrganismos gram-negativos entre os gêneros mais frequentemente cultivados em tecido profundo.

O nosso estudo conta com as limitações inerentes a um trabalho retrospectivo, porém traz uma contribuição à literatura ao analisar comparativamente culturas de espécimes profundos, coletadas em centro cirúrgico, em pacientes com lesões extensas e graves, estudando sua concordância, e também o número e as características dos microrganismos encontrados.

## CONCLUSÕES

Considerando-se o resultado das culturas realizadas a partir da coleta de diferentes espécimes profundos, em cerca de metade dos casos houve concordância total, com resultados idênticos entre as culturas de osso e de tendão em lesões tróficas profundas de extremidades. A discordância entre as culturas dos dois diferentes tecidos profundos (tendões e ossos) sugere que se faça, quando possível, a coleta de ambos os materiais.
